# Continuous and Unconstrained Tremor Monitoring in Parkinson's Disease Using Supervised Machine Learning and Wearable Sensors

**DOI:** 10.1155/2024/5787563

**Published:** 2024-05-20

**Authors:** Fernando Rodriguez, Philipp Krauss, Jonas Kluckert, Franziska Ryser, Lennart Stieglitz, Christian Baumann, Roger Gassert, Lukas Imbach, Oliver Bichsel

**Affiliations:** ^1^Rehabilitation Engineering Laboratory, Department of Health Sciences and Technology, ETH Zurich, Zurich, Switzerland; ^2^Department of Neurosurgery, University Hospital Zurich, University of Zurich, Zurich, Switzerland; ^3^Clinical Neuroscience Centre, University Hospital Zurich, University of Zurich, Zurich, Switzerland; ^4^Department of Neurosurgery, University Hospital Augsburg, Augsburg, Germany; ^5^Department of Neurology, University Hospital Zurich, University of Zurich, Zurich, Switzerland; ^6^Swiss Epilepsy Center, Klinik Lengg, Zurich, Switzerland

## Abstract

**Background:**

Accurately assessing the severity and frequency of fluctuating motor symptoms is important at all stages of Parkinson's disease management. Contrarily to time-consuming clinical testing or patient self-reporting with uncertain reliability, recordings with wearable sensors show promise as a tool for continuously and objectively assessing PD symptoms. While wearables-based clinical assessments during standardised and scripted tasks have been successfully implemented, assessments during unconstrained activity remain a challenge.

**Methods:**

We developed and implemented a supervised machine learning algorithm, trained and tested on tremor scores. We evaluated the algorithm on a 67-hour database comprising sensor data and clinical tremor scores for 24 Parkinson patients at four extremities for periods of about 3 hours. A random 25% subset of the labelled samples was used as test data, the remainder as training data. Based on features extracted from the sensor data, a Support Vector Machine was trained to predict tremor severity. Due to the inherent imbalance in tremor scores, we applied dataset rebalancing techniques.

**Results:**

Our classifier demonstrated robust performance in detecting tremor events with a sensitivity of 0.90 on the test-portion of the resampled dataset. The overall classification accuracy was high at 0.88.

**Conclusion:**

We implemented an accurate classifier for tremor monitoring in free-living environments that can be trained even with modestly sized and imbalanced datasets. This advancement offers significant clinical value in continuously monitoring Parkinson's disease symptoms beyond the hospital setting, paving the way for personalized management of PD, timely therapeutic adjustments, and improved patient quality of life.

## 1. Introduction

Neurological disorders are the globally leading source of disability, and among them, Parkinson's disease (PD) is the fastest-growing in prevalence, disability, and death [1]. Developing new therapies or improving existing ones may help address this growing challenge for healthcare systems and ultimately improve the lives of those affected by this condition. PD is a progressive, neurodegenerative disorder that presents with a broad spectrum of motor (mainly tremor, bradykinesia, and rigidity) and nonmotor symptoms. Affecting around 1% of the population over the age of 60, it is the second most common neurodegenerative pathology (after Alzheimer's Disease) [2] and causes significant decreases in the quality of life for those affected [3].

Accurately assessing the severity and frequency of PD symptoms is of distinct importance at almost all stages of disease management: diagnosis, monitoring disease progression, informing therapeutic course of action, and evaluating the effectiveness of treatment [4]. The gold-standard tool for PD assessment is the clinically validated International Parkinson's and Movement Disorder Society Unified Parkinson's Disease Rating Scale (MDS-UPDRS) [5]. Although reliable, UPDRS assessments are time-consuming to perform, require patients to be in-clinic, and only capture the patient's symptomatology during a brief period of time. PD symptoms heavily fluctuate, based on factors such as medication ON/OFF-state or behavioural context [6], and occasional assessments at any single point in time may fail to accurately reflect the overall disease state [7]. Alternatively, clinicians rely on patient self-reports in the form of “patient diaries.” The validity of these diaries, however, can be compromised by patients' lack of compliance, by patients not properly recognising symptoms within the clinical taxonomy understood by clinicians (e.g., dyskinesias), or by recall problems [8]. The quality of the diaries can also be affected by the decrease in cognitive capabilities that is highly prevalent at later stages of the disease [9]. This leads to a discrepancy between self-reported disease state and clinical UPDRS assessments, especially related to motor symptoms, that can be difficult to reconcile [10].

**Figure 1:**
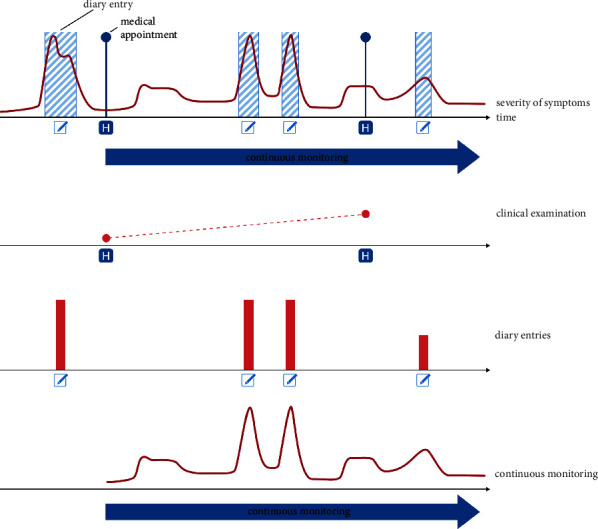
Sporadic clinical encounters do not provide an accurate portrayal of the disease course. Important bouts of severe symptoms, if they do not happen during clinical visits, might be missed. This leads to a gap between clinical assessment and patient self-report, which is the currently available system used to monitor the disease severity between appointments. This self-report, oftentimes in the form of diary entries, can however lack in precision, objectivity, and compliance on the side of the patient. Continuous monitoring would allow for automated and objective observation of disease state and progression with only moderate effort on the side of both clinicians and patients.

Wearable technology, through its potential to capture and characterise movement during both scripted tasks and in free-living environments, may be able to overcome some of the shortcomings of current PD assessment techniques—including the difficulty of recognising relevant symptoms if they present outside of the clinic, or the poor temporal resolution of MDS-UPDRS tests. This approach might provide clinicians with valuable information about symptom state outside what is observable during sporadic in-clinic encounters and patient self-reporting of symptoms (Figure 1), which can be particularly insightful for the common scenario of bouts of severe symptoms occurring at times during which a clinician is not present.

A number of groups have shown the feasibility of using wearable sensor data in combination with algorithms to accurately predict clinical scores during standardised tasks [11–20]. This has led to a body of literature devoted to a detailed characterisation of tremor in controlled experimental environments [18, 21, 22]. Nevertheless, symptom monitoring in free-living patients remains a challenge, with some methods having failed to deliver optimal results [23, 24]. In [25], the authors had no clinician-provided tremor labels to validate their system. In [13, 24], the only feedback signal available for algorithm training came from the aforementioned patient self-reporting. In [26], no ground-truth labels were available for algorithm training and validation outside of the clinical environment. To date, and to the best knowledge of the authors, no fully validated system to monitor clinical PD features in free-living environments exists [27–29].

In order to effectively tackle the transition from controlled environments to unconstrained, free-living patients, a significant challenge—from a signal processing perspective—stems from the fact that tremor (and other pathology-related movement patterns) cannot be easily isolated from sensor signals, especially when volitional movement is present at the same time. Machine learning techniques, due to their ability to isolate patterns from broadband, high-entropy signals, offer a potential solution to this problem. In this context, the use of unsupervised machine learning techniques that mine clinically relevant patterns from large amounts of wearable sensor data has been advocated [4]. Previous studies have employed several machine and deep learning approaches for wearable sensor-based approaches for monitoring Parkinsonian tremor, most often involving convolutional neuronal networks with raw data input, fast Fourier transform, or specific features (time and frequency domain) [18]. Others used approaches such as long short-term memory, dynamic neuronal network, multilayer perceptron, and gradient tree boost algorithm [18].

In this work, we bypass the need for self-supervised schemes by making direct use of clinician-provided labels gathered during continuous monitoring. We implement a cross-patient tremor-monitoring system that can be trained on a modestly sized and imbalanced dataset and use this method to investigate the problem of monitoring tremor fluctuations in free-living PD patients. To accomplish this, we rely on a 67-hour database of inertial measurement unit data manually labelled by clinicians using a custom-made mobile application, who were present during hours-long recording sessions during which patients went about their daily activities in a free-living environment. We derive features from the wearable sensor data based on accelerometry and gyroscopy and use these to train a machine learning algorithm that predicts tremor severity at each sensor location.

## 2. Methods

### 2.1. Dataset

Data collection was conducted with 24 patients at the Department of Neurology, University Hospital Zurich, with ethical approval granted by the cantonal ethics commission Zurich. Informed consent was obtained from all participants, who were randomly selected from those seeking inpatient refinement of their PD management, irrespective of age or gender, thus safeguarding an unbiased sample. Notably, patients generally represented more advanced stages of PD due to the nature of their hospital treatment. All subjects continued their prescribed PD medication regimen throughout the study to maintain their typical response to treatment during data capture (see Table 1). We did not have any dropouts.

The recording device consisted of inertial measurement units (IMUs) securely attached to the patients' wrists and ankles. The IMUs (ZurichMOVE) incorporated triaxial accelerometers and gyroscopes, recording movement at a sampling rate of 50 Hz. Sessions were designed to last approximately three hours (mean ± std. deviation: 2 h 48 min ± 1 h 5 min), capturing a broad range of voluntary movements and daily activities.

**Figure 2:**
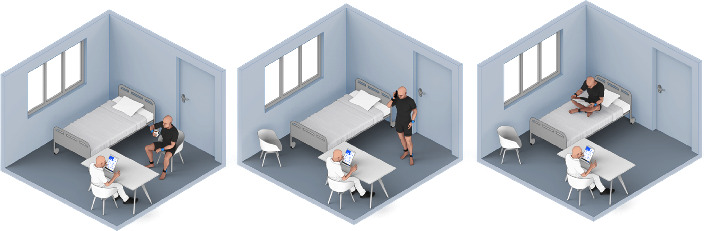
Depiction of various activities performed by patients during tremor assessment sessions. Clinicians conducted evaluations at three-minute intervals in an unconstrained environment simulating daily living scenarios, including reading, device usage, and light physical tasks.

Clinicians, present throughout the recording sessions, performed tremor assessments at three-minute intervals (duration mean ± std. deviation: 2.99 min ± 0.96 min), providing a rich temporal resolution of tremor data. This enabled the capture of tremor manifestations in a controlled yet representative environment, where patients engaged in various self-selected activities such as reading, writing, using electronic devices, and performing light physical tasks within the patient room, which mirrored a spectrum of daily life scenarios (see Figure 2).

Tremor severity for each limb was documented using a three-point scale: 0 indicating no tremor, 1 for mild tremor, and 2 for strong tremor. This bespoke categorisation was devised to streamline the complex clinical tremor evaluation typified by the UPDRS III, facilitating frequent, repeatable measurements conducive to our data analysis approach. A “no-data” entry was also available to mark periods where observation was not possible, such as personal privacy times.

The clinicians used a specialized application on a tablet that prompted them to record tremor scores every three minutes. The 30 seconds leading up to each score entry were extracted and segmented for detailed analysis, yielding a dataset comprising 4,850 tremor score-labelled samples across the three defined categories.

### 2.2. Data Preprocessing

**Figure 3:**
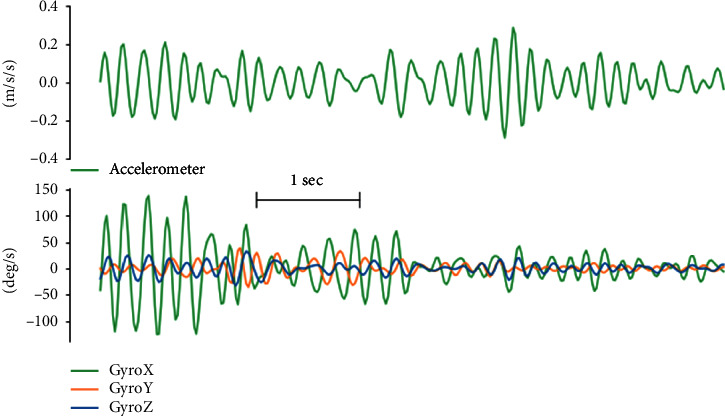
Preprocessed IMU data capturing tremor dynamics. The composite accelerometer signal, derived from the vector sum of three-axis data, is shown at the top, indicating fluctuating tremor magnitudes. Initially, the tremor intensity sharply peaks, diminishes briefly, rises again, and tapers off. Below, the triaxial gyroscope data reveal that rotational motion, particularly along the *X*-axis, is not consistently synchronized with these variations in tremor magnitude. The initial strong rotational activity is not mirrored during subsequent peaks of translational tremor, illustrating the distinct behaviours of these tremor components.

Parkinsonian tremor usually presents while the patient is at rest with frequencies in the 4–7 Hz band [30–32]. In order to isolate the tremor signal from the voluntary movement signal, both the accelerometer and gyroscope signals were high-pass-filtered (10th-order Butterworth filter, cutoff frequency of 3.5 Hz). In the interest of precision, the wearable sensors were configured to capture a voluntary-movement signal delineated by a specific frequency band. This was achieved by employing a 10th-order Butterworth bandpass filter with cutoff frequencies set between 0.5 and 3 Hz. While this methodological choice is primed for enhancing tremor signal fidelity, it may also encompass movement signatures potentially related to bradykinesia. This recognition, albeit indirect, suggests a promising avenue for further methodological advancements to explicitly characterise and classify bradykinesia within our computational framework. On the tremor signal, a low-pass filter (10th-order zero-phase Butterworth filter, cutoff frequency of 7.5 Hz) was also applied to eliminate higher frequency nontremor components, as well as high-frequency artefacts (Figure 3). Since only the oscillatory nature but not the directionality of movement is interesting for analysing tremor, only the magnitude of the filtered accelerometer vector was used.

To better capture the transient nature of tremor, a wavelet-based time-frequency analysis was performed. Employing the continuous wavelet transform, the frequency components representing tremor (between 3.5 and 7.5 Hz) were extracted into a time-dependant signal: with frequency *f*, time *t*, and wavelet coefficients *X*(*f*, *t*), and the resulting time-series *X*_tre_(*t*) was computed as *X*_tre_(*t*) = ∫_3.5 Hz_^7.5 Hz^*X*(*f*, *t*)d*f*. In summary, the preprocessing step consisted of extracting a total of 9 relevant time-series (Supp Table 1) from the 6 IMU channels (3 gyroscope channels + 3 accelerometry channels). All preprocessing steps and feature computations were performed in MATLAB (The MathWorks Inc., Natick, Massachusetts).

#### 2.2.1. Feature Extraction and Evaluation

From each of the preprocessed time-series, a number of features were derived (Supp Table 4 for non-wavelet-based time-series and Supp Table 5 for wavelet-based time-series). Roughly, these features can be categorised into the following three classes:

1. Time-domain statistics: General descriptive statistics that aimed at assessing the magnitude and variability of movement within a given timeframe. These include means, standard deviations, interquartile ranges, and variation coefficients.
2. Frequency-domain features: Features that capture the spectral characteristics of the signal (if tremor is present, signal power will concentrate in the 4–6 Hz frequency band). The power spectral density was estimated using Welch's method (8 segments, 50% overlap) [33, 34].
3. Nonlinear features: This set of features aims to quantify the regularity of a signal, which can be defined as the ability of past and future samples to predict the current one. Regularity measures, which go beyond describing signals in the time and frequency domains, have been proposed as better tools to capture the lack of ability to regulate movement that is characteristic in Parkinsonian tremor [32]. Generally, nonlinear approaches have been previously applied successfully to tremor detection and quantification [35, 36]. These features include approximate entropy and spectral entropy, as well as various autocorrelation-derived statistics.

Once the features for all time-series are computed, the effectiveness of each one as a predictor of tremor severity has to be evaluated. Each extremity was treated independently (i.e., right-hand tremor score only predicted with right-hand IMU-data). Although the overall dimensions of the feature space are 4850 samples x 90 features, not all of those 90 features are good predictors of clinical scores. To discard the ones that are not, a mutual information (MI) score is computed, which provides a good measure for the strength of the relationship between two variables [37].

Once the features were rated, only the 30 best performers were used in the subsequent steps of the analysis, and the remaining 60 features were discarded.

#### 2.2.2. Classifier and Sampling Strategies

We used a support vector machine (SVM) with radial basis functions (RBFs) for the classifier architecture given its ability to approximate nonlinear boundaries and train on a modestly sized dataset as well as its widespread use in the tremor-monitoring literature.

**Figure 4:**
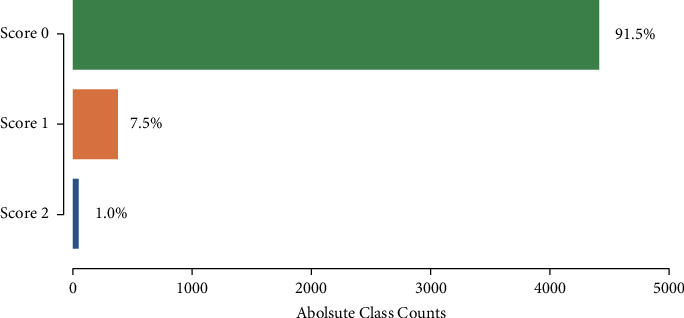
The dataset is highly imbalanced, with Score 0 (no tremor) representing 91.5% of all observations. Score 1 (mild tremor) represented only 7.5% of all observations and Score 2 (strong tremor) only 1%.

**Figure 5:**
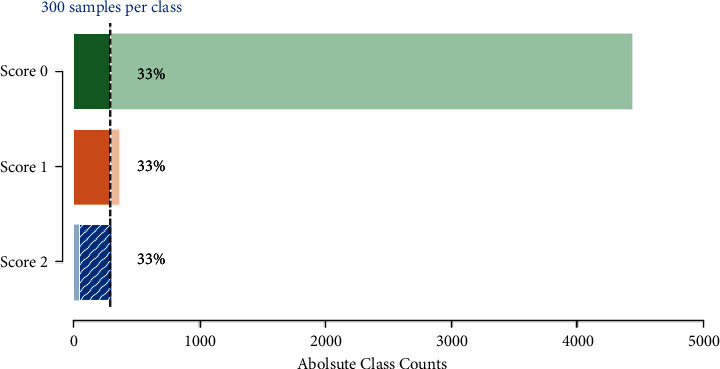
Balanced dataset after the resampling strategy. The diagonally striped, blue portion in Score 2 represents synthetic data.

Given the highly imbalanced tremor scores in our dataset (Figure 4), we had to use a resampling strategy to balance out the three classes. A balanced dataset was achieved by first applying random undersampling to the majority classes Score 0 and Score 1 until 300 samples were reached. To the minority class Score 2, we applied oversampling by creating new samples based on neighbouring points using the synthetic minority oversampling technique (SMOTE) [38] until 300 samples were reached (Figure 5).

The resampled dataset was subdued to a random 75/25 train-test split. All computations in feature space (evaluating features, building and training the classifier, and implementing sampling strategies) were performed in Python (Python Software Foundation, https://www.python.org/) making use of the specialized software packages scikit-learn [39] and imbalanced-learn [40].

## 3. Results

### 3.1. Selected Features

Using a best-in-class approach based on the MI-scores for the initial set of 90 features (for best and worst performances see Supp Tables 2 and 3), the 30 best performers are selected (full list and MI-Scores in Supp Table 6). These 30 features are subsequently used to train and test the classifier.

**Figure 6:**
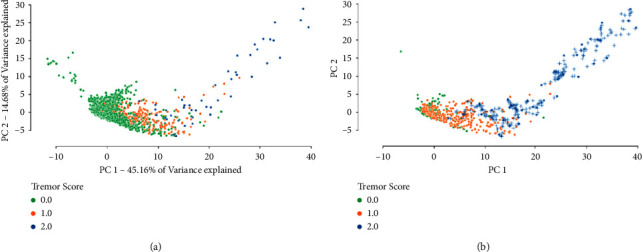
2D principal component analysis of (a) the initial dataset and (b) the final dataset after applying the resampling strategy. While in (a) the tremor scores are highly imbalanced, the proportions of the three classes have been equalised in (b). Synthetic data are represented with a “+” sign. PCA embedding is based on the 30 best-performing features in the initial dataset.

A two-dimensional *principal component analysis* of the initial dataset is computed (Figure 6(a)). The same feature transformation is applied to provide a visualisation of the resampled dataset (Figure 6(b)). Analogous to the rest of the analysis, these embeddings are constructed after the feature selection step (only making use of the 30 best features).

### 3.2. Classifier Performance

**Figure 7:**
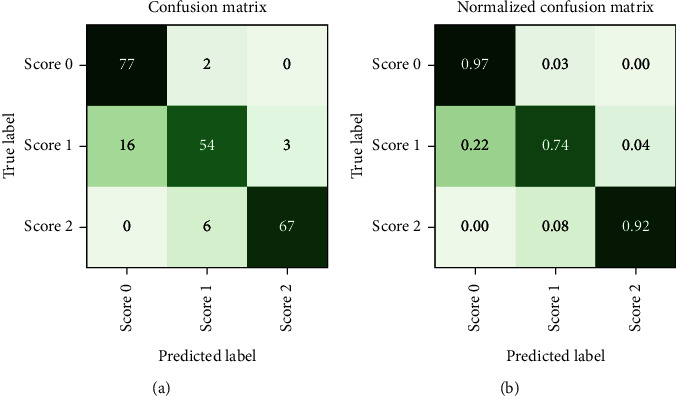
(a) Confusion matrix and (b) normalised confusion matrix for the test part of the *in-sample* dataset. Consistent with the principal component visualisations, there is a degree of overlap between the classes that results in some instances of adjacent classes being misclassified. The overall classification accuracy is 0.88.

After a random 75/25 train-test-split of the resampled dataset, the train portion contains 675 samples. After training, the remaining 225 samples in the test portion are used to evaluate the classification performance. The overall classification accuracy is 0.88 (198 out of 225 samples correctly classified). The associated confusion matrix (both absolute and normalised) is displayed in Figure 7. Sensitivity for samples containing tremor is 0.90. Accuracy scores are lowest for the intermediate class Score 1. There is a misclassification rate of 0.25 between the adjacent classes Score 0 and Score 1 and no misclassifications between nonadjacent classes Score 0 and Score 2.

**Figure 8:**
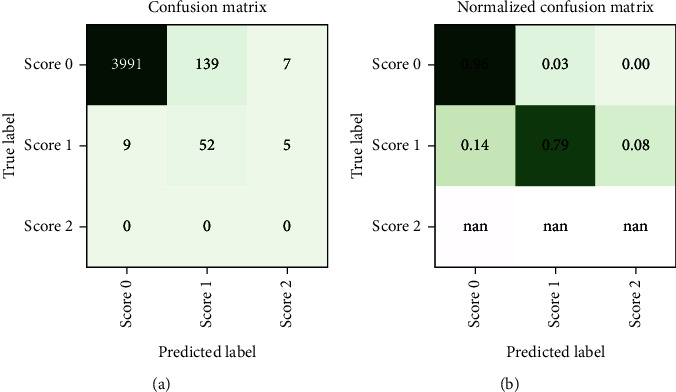
(a) Confusion matrix and (b) normalised confusion matrix for the out-of-sample predictions on the instances discarded during the undersampling technique. With an overall classification accuracy of 0.94, out-of-sample performance is comparable to in-sample performance.

Since neither training nor testing occurs on a dataset comprised of solely “real data” but both splits contain “synthetic instances” incorporated during the resampling process (about 28% of samples are synthetic), some further testing is performed to ensure that the classifier performs equally well when tested exclusively against “real data.” Due to the undersampling step, a large number of samples (4137 instances of Score 0 and 66 instances of Score 1) remain neither used for training nor for testing the classifier (although they were used in the feature selection step). Testing our classifier on these samples yields a classification accuracy of 0.94. The associated confusion matrices (absolute counts and normalised counts) are displayed in Figure 8. The performance on the discarded portion of the dataset (notwithstanding the fact that no instances of Score 2 are available for testing in this dataset) is comparable with the performance on the resampled dataset, with the same misclassification patterns being recognised in both—such as a mild tendency of Score 1 to be misclassified as Score 0.

## 4. Discussion

Accurately assessing the severity and frequency of fluctuating PD symptoms is of significant importance during all stages of PD management. In contrast to time-consuming clinical testing or sporadic patient-reporting, continuous wearables-based assessment might allow for continuous, objective, and automated tracking of PD symptoms.

### 4.1. Towards Parkinsonian Symptom Assessment during Unconstrained Activity

Miniaturised wearable sensors, in combination with modern signal processing techniques, show great promise as a tool for the continuous assessment of PD symptoms. While wearables-based clinical assessments during standardised and scripted tasks have been successfully implemented, assessments during unconstrained activity in free-living environments remain a challenge. In order to effectively transition from controlled environments to unconstrained, free-living patients, machine learning techniques that mine clinically relevant patterns from wearable sensor data need to be developed and validated.

Here, we developed and implemented a machine learning algorithm to assess tremor severity in free-living PD patients. We evaluated this system on a 67-hour database comprising wearable sensor data and clinical tremor scores from 24 PD patients, who wore an IMU on each extremity for periods of approximately 3 hours. Based on features extracted from the sensor data, a support vector machine (SVM) was trained to predict the clinician-logged tremor severity for each extremity. The classifier's ability to detect tremor was high (sensitivity of 0.90). Overall classification accuracy was 0.88 on the test-portion of the resampled dataset. To further test the robustness of the classifier, samples that had been previously discarded during the resampling strategy were classified. Here, overall accuracy was 0.94 and the results were comparable in terms of misclassification rates, suggesting that the classifier did not overfit the resampled dataset. In summary, we were able to implement a feature-based classifier for tremor monitoring that, with the utilisation of resampling techniques, can be trained even when only modestly sized and imbalanced datasets are available. This approach is especially valuable when large amounts of clinical data are not readily available or when gathering these data is costly.

### 4.2. Improving Parkinsonian Symptom Assessment in Free-Living Environments

Our tremor-classification system's lowest performance (accuracy = 0.74) was encountered when classifying Score 1, as it tends to be misclassified as an instance of class Score 0. A two-class approach (low tremor vs. high tremor severity) might still provide a more comprehensive picture of the severity of the Parkinsonian disease state between clinical visits as compared to currently available techniques, as well as provide more information about the timing and characteristics of tremor manifestations. Our successful use of resampling strategies supports the use of this family of techniques for processing wearable sensor data when the dataset is not large enough, or when the accompanying clinical scores are not balanced. However, validation of this tremor-classification system on previously unseen wearable sensor data is still lacking. While the current study provides valuable insights within its limitations, the potential for a more in-depth understanding of limb-specific tremors awaits future investigations with larger and more balanced datasets. In light of the chosen random 75/25 train-test split serving our specific resampling technique, we acknowledge the potential impact of this decision on result robustness. Future studies should consider exploring alternative validation methods, such as k-fold cross-validation, particularly in more balanced (a priori) datasets, to enhance the reliability and generalizability of predictive models. Despite tremor being a cardinal motor symptom of PD, other motor symptoms (especially bradykinesia) should be investigated and tested as well to assess Parkinsonian symptoms with wearable sensors in free-living patients.

Such a continuous monitoring with wearable sensors might allow to observe the course of the disease closely with only moderate effort. Wearables-based PD assessments may also be useful in other respects, for example, as more objective and accurate tools for evaluating new therapies—this could also streamline drug approval processes by improving both the quality and quantity of data gathered during a study, potentially reducing the required sample sizes [41]. While our tremor classification system focused on a frequency range of 4 to 7 Hz, this was chosen to mirror the frequencies most commonly observed in Parkinsonian resting tremor. However, we acknowledge the broader spectrum of tremor frequencies reported across varying studies and the individual variability among patients with Parkinson's disease. Future research could expand upon our findings by incorporating a wider frequency analysis, which may reveal more about the nuanced nature of tremor expressions in different subtypes of the disease. Such a tailored approach could lead to more personalized diagnostic and monitoring tools.

As we continue to refine the assessment of Parkinsonian symptoms within free-living environments, our findings suggest that personalized tremor frequency profiles could hold significant promise for advancing precision medicine in Parkinson's care. This aligns with the need for a more comprehensive understanding of tremor subtypes—resting, postural, and kinetic—and their specific clinical manifestations. Our current system sets a foundation for this advancement, with the potential to include subtype-specific tremor characteristics in subsequent iterations. This evolution in our system's capabilities could offer critical insights for developing individualized treatment strategies and enhancing the quality of life for those affected by PD.

The recognition of tremor subtypes—resting, postural, and kinetic tremors—is fundamental in the clinical assessment of Parkinson's disease (PD). Each subtype has distinctive characteristics and clinical implications that are critical for accurate diagnosis and treatment planning. Our tremor-classification system, while not designed to distinguish between these subtypes in its current iteration, lays the groundwork for future systems that could integrate subtype-specific features. This could further refine the monitoring and assessment of PD symptoms in free-living environments.

### 4.3. Expanding Applications beyond Parkinsonian Symptom Assessment

The utility of machine learning-based motor symptom classification systems transcends beyond parkinsonian symptom assessment in free-living patients. These computational models could be custom-tailored for patients with a diverse range of movement disorders, including essential tremor and gait disturbances, offering a versatile tool for symptom monitoring and personalized treatment strategies.

Moreover, postclassification tremor-related signals hold promise for enhancing therapeutic interventions. These signals could serve as integral components in the design of closed-loop deep brain stimulation systems, potentially refining the optimization of stimulation parameters. By dynamically responding to patients' fluctuating symptom severity, such systems could offer a more targeted and efficient therapeutic response.

Furthermore, the integration of these classified signals with neurofeedback methods offers a promising avenue for mitigating motor symptoms. Such techniques harness the power of real-time brain-computer interfaces, enabling patients to consciously control and reduce the manifestation of their symptoms, as demonstrated in [33]. These innovative applications underscore the transformative potential of machine learning in revolutionising symptom management for patients with movement disorders.

## 5. Conclusion

We implemented a tremor-classification system that can be trained on a modestly sized and highly unbalanced dataset. This machine learning implementation displayed a high accuracy (tremor detection sensitivity of 0.9 and overall classification accuracy of 0.88) for addressing the crucial problem of monitoring tremor fluctuations in free-living PD patients.

## Figures and Tables

**Table 1:** Demographic and clinical characteristics of the PD patients including age, sex, disease subtype, duration, stage (Hoehn–Yahr), motor examination scores (MDS UPDRS III ON/OFF), ON tremor score items 3.15–3.18, and daily levodopa equivalent dose (LED).

ID	Sex	Age	PD subtype	Disease duration (y)	Hoehn–Yahr scale	MDS UPDRS III on/off	3.15R, L; 3.16R, L; 3.17UR, UL, LR, LL;3.18	LED (mg/d)
PD1	M	67	Equivalent	8	2	22/42	0, 1; 0, 2; 0, 1, 0, 0; 1	1510
PD2	F	60	Akinetic-rigid	7	3	39/51	0, 0; 0, 1; 0, 0, 0, 0; 0	2050
PD3	F	80	Tremor	10	3	19/26	0, 0; 0, 0; 0, 0, 0, 0; 0	700
PD4	F	60	Tremor	5	3	17/32	1, 0; 1, 0; 1, 0, 1, 0; 3	625
PD5	M	61	Equivalent	15	3	18/53	0, 0; 0, 0; 0, 0, 1, 1; 1	630
PD6	M	67	Equivalent	11	4	67/72	3, 1; 3, 1; 3, 0, 0, 0; 4	1230
PD7	F	73	Equivalent	7	2	16/39	0, 0; 0, 0; 0, 0, 0, 0; 0	1465
PD8	M	51	Equivalent	4	2	31/45	1, 1; 1, 1; 1, 0, 0, 0; 1	1540
PD9	M	67	Equivalent	12	2	44/57	0, 1; 0, 1; 0, 0, 0, 0; 0	560
PD10	F	65	Akinetic-rigid	13	3	33/52	1, 0; 1, 0; 1, 0, 0, 0; 0	510
PD11	F	54	Akinetic-rigid	9	3	13/22	1, 0; 0, 0; 0, 0, 0, 0; 1	910
PD12	F	57	Equivalent	9	3	20/55	4, 1; 1, 0; 4, 1, 2, 0; 4	870
PD13	F	56	Tremor	6	2	9/33	0, 0; 0, 0; 0, 0, 0, 0; 0	1350
PD14	F	62	Tremor	14	4	55/n.a.	3, 2; 1, 2; 1, 2, 2, 1; 2	500
PD15	M	73	Akinetic-rigid	23	2	39/62	0, 0; 0, 0; 0, 0, 0, 0; 0	1290
PD16	F	87	Equivalent	9	4	50/n.a.	2, 1; 0, 0; 2, 1, 2, 1; 4	620
PD17	F	53	Akinetic-rigid	17	3	23/n.a.	0, 0; 0, 0; 0, 0, 0, 0; 0	510
PD18	M	57	Tremor	8	2	19/23	2, 0; 1, 1; 3, 0, 0, 0; 3	60
PD19	M	52	Akinetic-rigid	17	2.5	15/24	0, 0; 0, 0; 0, 0, 0, 0; 0	1430
PD20	M	64	Tremor	6	3	21/38	1, 0; 1, 1; 0, 0, 0, 0; 0	920
PD21	M	68	Tremor	7	3	33/50	1, 0; 1, 1; 2, 0, 0, 0; 3	750
PD22	M	71	Tremor	4	2.5	23/36	0, 1; 0, 1; 0, 3, 0, 0; 3	1100
PD23	M	72	Equivalent	11	2	18/n.a.	0, 0; 1, 1; 0, 0, 0, 0; 0	350
PD24	F	69	Equivalent	8	3	18/28	0, 0; 0, 0; 0, 1, 0, 1; 1	1740

## Data Availability

The data used to support the findings of this study are not publicly available due to ethical restrictions.

## Supplementary Materials

Supplementary Materials Table 1: time series computed during preprocessing step. Table 2: best performing features. ^*∗*^Mutual-Information score, one for each channel. ^*∗∗*^Some features can perform well in some channels and poorly in others. Here, only the best-performing channels are displayed (ordered accordingly). Table 3: worst performing features. ^*∗*^Mutual-Information score, one for each channel. ^*∗∗*^Some features can perform well in some channels and poorly in others. Here, only the best-performing channels are displayed (ordered accordingly). Table 4: list of comprehensive features. ^*∗*^nAR stands for normalised autocorrelation. Table 5: list of reduced features. Table 6: selected features, ranked by MI-Score.
