# Supplementary material for: Continuous and Unconstrained Tremor Monitoring in Parkinson's Disease Using Supervised Machine Learning and Wearable Sensors
Source: Parkinsons Dis. 2024 May 20;2024:5787563. doi: 10.1155/2024/5787563 (PMC11129907; doi:10.1155/2024/5787563)
Supplement: Supplementary Materials — Table 1: time series computed during preprocessing step. Table 2: best performing features. ∗Mutual-Information score, one for each channel. ∗∗Some features can perform well in some channels and poorly in others. Here, only the best-performing channels are displayed (ordered accordingly). Table 3: worst performing features. ∗Mutual-Information score, one for each channel. ∗∗Some features can perform well in some channels and poorly in others. Here, only the best-performing channels are displayed (ordered accordingly). Table 4: list of comprehensive features. ∗nAR stands for normalised autocorrelation. Table 5: list of reduced features. Table 6: selected features, ranked by MI-Score. [file 5787563.f1.zip › STab1.pdf]

Timeseries	Description
AccelTremor	Magnitude of bandpass-filtered (3.5-7.5 Hz) accelerometer channels
AccelVoluntary	Magnitude of lowpass-filtered (3 Hz) accelerometer channels
GyroXTremor	Bandpass-filtered (3.5-7.5 Hz) gyroscope X-channel
GyroYTremor	Bandpass-filtered (3.5-7.5 Hz) gyroscope Y-channel
GyroZTremor	Bandpass-filtered (3.5-7.5 Hz) gyroscope Z-channel
AccelWavelets	Integral over tremor-frequency-range for wavelet coefficients of accelerometer magnitude
GyroXWavelets	Integral over tremor-frequency-range for wavelet coefficients of gyroscope X-channel
GyroYWavelets	Integral over tremor-frequency-range for wavelet coefficients of gyroscope Y-channel
GyroZWavelets	Integral over tremor-frequency-range for wavelet coefficients of gyroscope Z-channel
