# Supplementary material for: Continuous and Unconstrained Tremor Monitoring in Parkinson's Disease Using Supervised Machine Learning and Wearable Sensors
Source: Parkinsons Dis. 2024 May 20;2024:5787563. doi: 10.1155/2024/5787563 (PMC11129907; doi:10.1155/2024/5787563)
Supplement: Supplementary Materials — Table 1: time series computed during preprocessing step. Table 2: best performing features. ∗Mutual-Information score, one for each channel. ∗∗Some features can perform well in some channels and poorly in others. Here, only the best-performing channels are displayed (ordered accordingly). Table 3: worst performing features. ∗Mutual-Information score, one for each channel. ∗∗Some features can perform well in some channels and poorly in others. Here, only the best-performing channels are displayed (ordered accordingly). Table 4: list of comprehensive features. ∗nAR stands for normalised autocorrelation. Table 5: list of reduced features. Table 6: selected features, ranked by MI-Score. [file 5787563.f1.zip › STab2.pdf]

Feature	MI-Scores*	Channels**	Feature Type
First Autocorrelation Peak	0.096, 0.085	AccelTremor, GyroXTremor	Non-linear
Spectral Entropy	0.094, 0.087, 0.084	AccelTremor, GyroXTremor, GyroYTremor	Non-linear
Interquartile Range	0.082	GyroYTremor	Time-domain
Freq. Spectrum: Std. Deviation	0.08, 0.078, 0.072	GyroXTremor, GyroYTremor, AccelTremor	Frequency-domain
