# Supplementary material for: Continuous and Unconstrained Tremor Monitoring in Parkinson's Disease Using Supervised Machine Learning and Wearable Sensors
Source: Parkinsons Dis. 2024 May 20;2024:5787563. doi: 10.1155/2024/5787563 (PMC11129907; doi:10.1155/2024/5787563)
Supplement: Supplementary Materials — Table 1: time series computed during preprocessing step. Table 2: best performing features. ∗Mutual-Information score, one for each channel. ∗∗Some features can perform well in some channels and poorly in others. Here, only the best-performing channels are displayed (ordered accordingly). Table 3: worst performing features. ∗Mutual-Information score, one for each channel. ∗∗Some features can perform well in some channels and poorly in others. Here, only the best-performing channels are displayed (ordered accordingly). Table 4: list of comprehensive features. ∗nAR stands for normalised autocorrelation. Table 5: list of reduced features. Table 6: selected features, ranked by MI-Score. [file 5787563.f1.zip › STab3.pdf]

**Feature**	**MI-Scores***	**Channels****	**Feature Type**
Variation Coefficient	0.00, 0.00, 0.003	GyroXTremor, GyroZTremor, GyroYTremor	Time-domain
Data Range	0.003, 0.014	AccelVoluntary, GyroZWavelets	Time-domain
Sample skewness	0.003, 0.023	AccelVoluntary, GyroZWavelets	Time-domain
Freq. Spectrum: Mean	0.003, 0.015, 0.015	AccelTremor, GyroYTremor, GyroXTremor	Frequency-domain
