# Supplementary material for: Continuous and Unconstrained Tremor Monitoring in Parkinson's Disease Using Supervised Machine Learning and Wearable Sensors
Source: Parkinsons Dis. 2024 May 20;2024:5787563. doi: 10.1155/2024/5787563 (PMC11129907; doi:10.1155/2024/5787563)
Supplement: Supplementary Materials — Table 1: time series computed during preprocessing step. Table 2: best performing features. ∗Mutual-Information score, one for each channel. ∗∗Some features can perform well in some channels and poorly in others. Here, only the best-performing channels are displayed (ordered accordingly). Table 3: worst performing features. ∗Mutual-Information score, one for each channel. ∗∗Some features can perform well in some channels and poorly in others. Here, only the best-performing channels are displayed (ordered accordingly). Table 4: list of comprehensive features. ∗nAR stands for normalised autocorrelation. Table 5: list of reduced features. Table 6: selected features, ranked by MI-Score. [file 5787563.f1.zip › STab4.pdf]

Feature	Acronym	Feature type
Root mean square value	RMS	Time-domain
Data range	range	Time-domain
Inter-quartile range	IQR	Time-domain
Variation coefficient	varCoeff	Time-domain
Sample skewness	skew	Time-domain
Energy at peak	Epeak	Frequency-domain
Proportion of Energy at peak	Eprop	Frequency-domain
Spectrum mean frequency	specMean	Frequency-domain
Spectrum standard deviation	specStd	Frequency-domain
Sample approximate entropy	sampleEnt	Non-linear
Spectral entropy	specEnt	Non-linear
First nAR* peak	pk1	Non-linear
Second nAR peak	pk2	Non-linear
Peak difference	pkDiff	Non-linear
nAR peak decay (exponential fit)	pkDecay	Non-linear
Root mean square nAR	AutoRMS	Non-linear
