# Supplementary material for: Continuous and Unconstrained Tremor Monitoring in Parkinson's Disease Using Supervised Machine Learning and Wearable Sensors
Source: Parkinsons Dis. 2024 May 20;2024:5787563. doi: 10.1155/2024/5787563 (PMC11129907; doi:10.1155/2024/5787563)
Supplement: Supplementary Materials — Table 1: time series computed during preprocessing step. Table 2: best performing features. ∗Mutual-Information score, one for each channel. ∗∗Some features can perform well in some channels and poorly in others. Here, only the best-performing channels are displayed (ordered accordingly). Table 3: worst performing features. ∗Mutual-Information score, one for each channel. ∗∗Some features can perform well in some channels and poorly in others. Here, only the best-performing channels are displayed (ordered accordingly). Table 4: list of comprehensive features. ∗nAR stands for normalised autocorrelation. Table 5: list of reduced features. Table 6: selected features, ranked by MI-Score. [file 5787563.f1.zip › STab6.pdf]

Feature	MI-Score
AccelTremor: pk1	0.096
AccelTremor: specEnt	0.094
GyroXTremor: specEnt	0.087
AccelTremor: AutoRMS	0.087
GyroXTremor: pk1	0.085
GyroYTremor: specEnt	0.084
GyroYTremor: IQR	0.082
GyroYTremor: pk1	0.082
GyroXTremor: specStd	0.08
GyroYTremor: specStd	0.078
GyroYTremor: AutoRMS	0.073
AccelTremor: specStd	0.073
GyroYTremor: pk2	0.068
GyroXTremor: pk2	0.068
GyroXTremor: AutoRMS	0.067
GyroZtremor: pk1	0.066
GyroZTremor: specEnt	0.065
GyroZTremor: pk2	0.065
AccelTremor: pk2	0.065
AccelTremor: IQR	0.064
GyroYTremor: Epeak	0.064
AccelTremor: Eprop	0.064
GyroXTremor: IQR	0.063
GyroZTremor: specStd	0.062
AccelTremor: RMS / AccelVol: RMS	0.061
GyroZTremor: IQR	0.06
AccelTremor: Epeak	0.06
GyroXTremor: Epeak	0.06
GyroYWavelets: IQR	0.056
GyroXWavelets: IQR	0.054
