# Supplementary material for: Continuous and Unconstrained Tremor Monitoring in Parkinson's Disease Using Supervised Machine Learning and Wearable Sensors
Source: Parkinsons Dis. 2024 May 20;2024:5787563. doi: 10.1155/2024/5787563 (PMC11129907; doi:10.1155/2024/5787563)
Supplement: Supplementary Materials — Table 1: time series computed during preprocessing step. Table 2: best performing features. ∗Mutual-Information score, one for each channel. ∗∗Some features can perform well in some channels and poorly in others. Here, only the best-performing channels are displayed (ordered accordingly). Table 3: worst performing features. ∗Mutual-Information score, one for each channel. ∗∗Some features can perform well in some channels and poorly in others. Here, only the best-performing channels are displayed (ordered accordingly). Table 4: list of comprehensive features. ∗nAR stands for normalised autocorrelation. Table 5: list of reduced features. Table 6: selected features, ranked by MI-Score. [file 5787563.f1.zip › Supp Table Legends.docx]

Table 1: Timeseries computed during pre-processing step

Table 2: Best performing features. (*) Mutual-Information score, one for each channel.

(**) Some features can perform well in some channels and poorly in others. Here, only the best-performing channels are displayed (ordered accordingly).

Table 3: Worst performing features. (*) Mutual-Information score, one for each channel.

(**) Some features can perform well in some channels and poorly in others. Here, only the best-performing channels are displayed (ordered accordingly).

Table 4: List of comprehensive features. (*) nAR stands for normalized auto-correlation.

Table 5: List of reduced features

Table 6: Selected features, ranked by MI-Score
